# Fear and rumours regarding placental biopsies in a malaria-in-pregnancy trial in Benin

**DOI:** 10.1186/s12936-018-2578-9

**Published:** 2018-11-15

**Authors:** Adélaïde Compaoré, Susan Dierickx, Fatou Jaiteh, Alain Nahum, Towanou Francis Emmanuel Bohissou, Halidou Tinto, Susana Scott, Umberto D’Alessandro, Henk Schallig, Koen Peeters Grietens

**Affiliations:** 1 Clinical Research Unit of Nanoro, Institut de Recherche en Sciences de la Santé, Nanoro, Burkina Faso; 2 Medical Anthropology Unit, Department of Public Health, Institute of Tropical Medicine, Antwerp, Belgium; 3 Centre of Expertise on Gender, Diversity and Intersectionality (RHEA), Vrije Universiteit Brussel, Pleinlaan 2, 1050 Brussels, Belgium; 4 Centre for Research on Culture and Gender, Ghent University, Rozier 44, 9000 Ghent, Belgium; 5 Amsterdam Institute of Social Science Research, Amsterdam, The Netherlands; 6 Medical Research Council Unit The Gambia at London, School of Tropical Medicine and Hygiene, Fajara, The Gambia; 7 Centre de Recherches Entomologiques de Cotonou, Cotonou, Benin; 8 London School of Tropical Medicine and Hygiene, London, UK; 9 Department of Medical Microbiology-Parasitology Unit, Academic Medical Centre, Amsterdam, 1105 AZ The Netherlands; 10 Partners for Applied Social Sciences (PASS) International, Tessenderlo, Belgium; 11 School of Tropical Medicine and Global Health, Nagasaki University, Nagasaki, Japan

**Keywords:** Rumour, Placenta, Clinical trial, Community health worker, Pregnancy, Malaria

## Abstract

**Background:**

A multi-country, community-based trial on scheduled screening and treatment for malaria in pregnancy was conducted in Benin, The Gambia and Burkina Faso. Despite standardized procedures and outcomes, the study became subject to rumours and accusations of placenta being sold for mystical and financial gain by trial staff, leading to drop-out rates of 30% and the consequent halting of placental biopsy sampling in Benin. This paper explores the role of socio-cultural beliefs related to placenta and identified additional factors contributing these rumours.

**Methods:**

A qualitative comparative emergent-theory design was used to assess social factors related to trial implementation and uptake in the three countries. Data from participant observation, informal conversations, group discussions and interviews were triangulated and analysed with NVivo Qualitative Analysis software.

**Results:**

Despite similar sociocultural beliefs about the sacred nature of the placenta in all three study countries, these beliefs did not affect participation rates in Burkina Faso and The Gambia and placenta-related rumours only emerged in Benin. Therefore, the presence of beliefs is not a sufficient condition to have generated placenta-selling fears. The rumours in Benin reflected the confluence of placenta-related beliefs and factors related to the implementation of the trial (including a catalysing adverse event and miscommunication during the informed consent procedure). Furthermore, distinct socio-political factors contributed to the emergence of rumours, including the historical distrust in governmental organizations and the tense relationship between some of the actors involved in the trial.

**Conclusion:**

Transdisciplinary social science research designs should accompany the implementation of the trial. The integration of multiple stakeholders’ knowledge and involvement is required to define and solve upcoming barriers.

## Background

*I have one of my aunts in the village here. Her daughter*-*in*-*law gave birth in the village. After the delivery, they [the health care providers] said that they will cut the placenta. She wasn’t aware of anything! The doctors had come, and they said they will cut her placenta! This is where they started their work, and now the child is dead! So, that day, there was an uprising of the whole village. Delegates and elderly people from the village were on the scene! This is when everyone refused. No one will cut the placenta of our children anymore.* (Adult woman, group discussion, Benin).

Enrolling study participants in a clinical or community-based trial often represents a dilemma for community members as participation is associated to both perceived benefits and risks that they are required to outweigh [1–3]. In resource-limited settings, trial participation may offer access to enhanced medical and nursing care, but at the same time is often met with suspicion and fear [4]. A commonly reported perceived disadvantage of participating in medical research lies in the necessity of the collection of blood or other bodily substances [2, 5]. Since colonial times, fears about body parts trade, blood-stealing, sterilization campaigns and the deliberate spreading of diseases in relation to public health interventions have been documented [4, 6–9]. The presence of rumours can undermine research ethics and influence the relationship between communities and research institutions [10, 11]. It has been shown that rumours regarding the collection of blood and bodily substances shape the conduct and outcome of medical research [7], disrupt recruitment and retention of study participants and potentially lead to research interruption [4, 5, 12]. In Zambia, during a malnutrition trial, mothers feared that the body parts of their children had been taken and sold by the trial team [4]. In The Gambia, recruitment was ended in a village due to concerns regarding blood-taking during a malaria treatment trial [6]. Another example is the paper published by Pool et al. [13] describing how the study procedure in Manhiça (measuring a child, giving drinks and biscuits to the mother…) has been interpreted as an expectation of a child’s death.

Rumours can also arise due to placenta biopsies which are frequently collected to diagnose malaria in pregnancy by histology [14]. From a biomedical perspective, the placenta represents a specialized pregnancy organ responsible for essential functions, including fetal support, nourishment and protection [15]. At the sociocultural level, the representation of the placenta varies according to societies. In many regions of the world, including West Africa, the afterbirth is subject to a range of continuously evolving beliefs and practices. In Nigeria, for example, the placenta is used to induce labour in traditional obstetric medicine [16]. In China, women profit from selling their placenta to pharmacies as the organ is used as a powerful traditional Chinese medicine [17]. In high-income countries, such as the United States and Canada, some homebirth mothers, consume placenta in order to improve their mood and have general health benefits [18].

This anthropological study was ancillary to the cluster-randomized trial ‘Community-based scheduled screening and treatment of malaria in pregnancy for improved maternal and infant health: a cluster-randomized trial in The Gambia, Burkina Faso and Benin’ (EU FP7 funded project, clinicalTrial.gov: NCT01941264 (10 September 2013), referred to hereafter as ‘COSMIC’) [19]. The main objective of COSMIC was to develop a low-cost intervention in resource poor countries. The project wanted to determine the added value of community scheduled screening and treatment (CSST) of pregnant women (as compared to IPTp-SP alone implemented in health facilities) implemented through the community health workers (CHW) involved in community case management of malaria (CCMm). One of the trial outcomes was placental malaria which was measured by the collection of placenta biopsies. Between November 2013 and November 2015, the trial recruited a total of 4731 pregnant women across the three study sites (Table 1). While The Gambia and Burkina Faso recruited the required 1800 women, Benin recruited only 54% (971/1800) of the targeted sample with a loss to follow-up of 29.9% (290/971) due to rumours surrounding placenta biopsies at delivery.

**Table 1:** Lost to follow-up of pregnant women in COSMIC trial per study site (N = 4731)

	Burkina Faso (N = 1800)		The Gambia (N = 1960)		Benin (N = 971)	
	n	%	n	%	n	%
Participants lost to follow-up	62	3.4	113	5.8	290	29.9

This study explores the reasons why—despite similar outsets and trial procedures—rumours emerged in Benin, leading to the premature ending of placental biopsies collection. This comparative study allows a better understanding of how medical research can be challenged by rumours and identify the reasons behind the emergence of rumours beyond “social and cultural beliefs”.

## Methods

### Study design

Anthropological research was conducted in the three trial countries to assess social factors related to trial implementation and uptake. A qualitative comparative emergent-theory design was used, triangulating data from in-depth interviews, group discussions and participant observation, including informal conversations.

### Study site and population

#### Benin

The anthropological study was carried out in 20 rural communities (15 intervention and 5 control villages) in Zinvié and Zè districts, in the Atlantic Region of South Benin. The population consists mainly of the Aizo ethnic group who live mainly from subsistence farming, producing mostly manioc, palm oil and pineapple. The most commonly practiced religions are Catholicism, *Christianisme céleste* (a new Christian church originating from Porto Novo in 1947) [20] and the animist *voodoo* belief system [21].

The governmental health system in Benin has a pyramidal structure and is divided into three levels. At the central level, there is the National Referral Hospital, at the intermediate level the Departmental hospital and at the peripheral level, there are the Zonal referral hospital, the Commune Health Centres and the Arrondissement Health Centres. In addition to governmental health centres, the health system in Benin comprises private and denominational health centres in urban as well as rural areas.

#### The Gambia

Fieldwork was conducted in 7 intervention and 4 control villages of the Upper River Region. The population belonged mainly to the Mandinka, Serahuli and Fulani ethnic groups, with the majority practicing Islam. Village inhabitants mostly lived of subsistence farming of groundnut maize, sorghum, cattle rearing and remittances received from relatives who migrated to urban parts of The Gambia or abroad.

#### Burkina Faso

Fieldwork was carried out in the rural department of the central-western region of the country, in the Nanoro health district, located at 85 km from the capital city Ouagadougou. The anthropological study was conducted in 12 intervention and 1 control villages. The population is rural, predominantly of Mossi ethnic group and practicing Islam. They rely on subsistence farming and animal husbandry [22].

### The objectives and procedures of the COSMIC trial

The COSMIC trial aimed to establish whether maternal and infant health can be improved by implementing CSST of malaria among pregnant women by CHWs [19]. Pregnant women in their second trimester were identified and enrolled by trained study nurses in the trial during their first antenatal care (ANC) visits where they received their first dose of intermittent preventive treatment with sulfadoxine-pyrimethamine (IPTp-SP). Community consent was obtained after sensitization meetings and pregnant women provided individual signed informed consent (by thumb print and the signature of an independent witness in case of illiteracy). An assent form was completed for pregnant women younger than 16 years of age (in The Gambia) or not considered as an adult (i.e. under 18 and not married in Burkina Faso and Benin).

After inclusion in the trial, women in the intervention arm were followed up at home on a monthly basis by CHWs and encouraged to attend ANC for subsequent IPTp-SP. During these visits, a malaria rapid diagnostic test (RDT) was performed by the CHWs and positive women were treated with artemether-lumefantrine (AL). All women (intervention and control arm) were encouraged to deliver at health facilities in order to collect blood samples for haemoglobin measurement and to detect malaria just before delivery. A placenta biopsy was collected at delivery and the new-born was examined. For women not delivering in a health facility, study nurses visited them whenever possible at home to collect the same information and biological samples as obtained at health facilities.

### Data collection

#### Participant observation and informal conversations

Participant observations consisted of participating in everyday activities in the communities and observing the implementation of the trial. Reiterated informal conversations with community members were carried out both with women who participated and women who did not participate. The informal nature of these methods leads to an in-depth understanding of sensitive topics such as witchcraft, rumours and trust in the research team.

#### Interviews

Interviews were conducted with community members and health staff of the study team (Table 2). No formal interviews were done with participants who refused to participate in Benin because of the precarious situation. Interviews were recorded and fully transcribed. When not possible or inappropriate, given the sensitive nature of the topic discussed, the conversation was not recorded but the content was written down immediately after the interview. Interviews were mostly carried out in the local languages.

**Table 2:** Overview of collected data

Participants	Interviews	Group discussions
*Participants Benin*		
Women	25	4
Men	3	1
Village chiefs	11	0
Traditional healers	4	0
Midwives	1	0
Community health workers	19	0
Research team members	7	0
Total Benin	70	5
*Participants Burkina Faso*		
Women	14	5
Community health workers	13	0
Total Burkina Faso	27	5
*Participants The Gambia*		
Women	9	3
Men	4	2
Traditional birth attendants	3	0
Community health workers	4	0
Village chiefs	6	0
Total The Gambia	26	5

#### Group discussions

Group discussion were recorded and fully transcribed (Table 2). The participation of different members often enriched the data by providing different points of view in a discussion.

### Sampling

Following the principle of gradual selection, initial sampling in all three countries was purposive. As the study developed, informants were theoretical selected (in accordance with emerging results/theory) and categorized in relation to relevant criteria (e.g. gender, age, socio-economic status and decision to participate). In order to increase confidentiality with respondents and consequent reliability of the data, snowball sampling—sampling using participants to identify additional respondents—was used.

### Data analysis

In accordance with the research strategy, preliminary data from the observations, informal conversations, interviews and group discussions were collected and analysed to inform the question guide; further data collection was then conducted to confirm or refute temporary results until saturation was reached. Data were systemized and analysed with NVivo Qualitative Analysis software (QSR International Pty Ltd. Cardigan UK). Illustrative quotes are presented in Table 3 and are selected based on the richness of information on a particular topic presented in the text.

**Table 3:** Illustrative quotes from research in Benin

*Description and progression of the rumours*
She has heard people say that as soon as you go to a health centre for care, there are some people who will receive you. They will take your name and will make you sign a paper. After that they will take some of your blood. And as soon as you give birth, they will cut the placenta of your child and analyse it. They will wait until the child is five years old. After these five years, the child will die. (…) We refused that. (Community member, woman, interview)
They have formed an association. In this association, if a woman agrees, after childbirth, a bit of the child’s placenta will be cut off and analysed. (…) Our parents have never done these kinds of analyses to us. Our parents never did that! That’s why, when they came here, we said we will kill them and sent them all away. (Community member, woman, group discussion)
*Placenta beliefs and rituals*
The placenta means the rope through which the child comes into the world. Each ‘koukin’ has its ritual according to the day the child was born on and these are things that must be respected. Otherwise it affects the child. (Traditional chief, man, interview)
After childbirth, so that the child is well loved by his family’s members, one does not immediately bury his placenta. It is hung somewhere in the house and its smell evaporates throughout the household. There, the child is loved by all the members of his family. Wherever he goes in this family he will be loved by everyone and we will know that he is really born from this family. (Community member, man, group discussion)
*Implementation of informed consent procedures*
…This is related to life and death. The placenta is the part of death. If we do not give the part of death now, immediately death will hit the child and therefore the woman. That’s why we have to give a part to death…Do not use vulgar words to talk about this (placenta)! When you say “koukan, e nan kan”, this means “we will cut the placenta”. But we do not cut… You want to take the part of death. So instead of saying “e nan kan” (…) we must find the subtler words. (Researcher, man, informal conversation)
I cannot accept that my wife is called in secret to raise awareness. Worse still, when these women go into consultation, they are given 20.000 CFA [35 USD] in secret, and they are now forced to join the project. It was the straw that broke the camel’s back. Our health centre has been closed for at least three months, and it is now that it started working. So, it’s like a conspiracy between midwives and pregnant women. If it’s like that, there’s enough to suspect bad intentions. (Community member, man, informal conversation)
*The political involvement of CHWs*
It means that the CHW also forget some things; man should not put many things in the nostrils; it will suffocate them, and yet: that’s their problems. I explain that to them because, politics spoils a lot of things. Politics spoils a lot of things. A lot, you cannot imagine. You have been chosen to prick pregnant women and to draw blood, and also to give medicine to children, and you put yourself in politics… There are people who have even said that vaccinators will not come to their homes because those are in the opposition and what will they [vaccinating agents] do in their homes? They will put strange things in their children’s mouths. (Village chief, man, interview)
*Health system factors*
The vaccinations we give to children are free things. We do not pay before making these vaccinations and yet, there are some people who refuse that because when children are vaccinated like that, they start having illnesses and parents spend a lot finding treatment; That’s why there are people who refuse things that are given for free… (Community member, man, group discussion)
Last time, vaccinators went from house to house to vaccinate my children. A few days later, these children all became ill. I took them all to the hospital to be cared for. I arrived in the centre, but no one was interested in us. They even told me to take my children home, that they do not even know where we got these diseases. I was really unhappy that day since it is they who sent us these agents to vaccinate our children. And why, if there is afterwards a problem, and we come to see them, they refuse to take us? We cannot accept these kinds of things. That’s why, if there is a project that comes, and we are not informed, we take our guard to not fall into their traps. (Community member, man, informal conversation)
In health centres, in maternity ward, what happens? It’s just like, since COSMIC people have invaded their work, their business does not work as before; I did not understand why; because there is a medicine there, that COSMIC people give to pregnant women free of charge. And this medicine is also available in health centres. It seems to me that they sell these drugs. (Village chief, man, interview)

### Ethics

This anthropological study was approved by the *Comité National d’Ethique pour la Recherche en Santé* in Benin (number 059/MS/DC/SGM/DFR/CNERS/SA), the *Comité d’Ethique du Centre Muraz et le Ministère de la Santé* (CERS) in Burkina Faso (number 017-2014/CE-CM); MRC Scientific Coordinating Committee and The Gambia Government/MRC joint Ethics Committee (SCC number 1335); and by the Institutional Review Board of the Institute of Tropical Medicine, Antwerp, Belgium. The interviewers followed the Code of Ethics of the American Anthropological Association (AAA) [23]. All interviewees were informed before the start of the interview about project goals, the topic and type of questions as well as their right to decline participation or to interrupt the conversation at any time. Anonymity was guaranteed and confidentiality of interviewees assured by assigning a unique code number to each informant. Verbal instead of written consent was preferred as requesting the subject’s signature could have been a potential reason for mistrust.

## Results

### Description and progression of the rumours

#### The onset of rumours

In Benin, soon after the start of the trial, people started accusing the research team of selling trial participants’ placenta for financial gain and/or for sorcery purposes in the benefit of the trial staff themselves or the unknown buyers of the placenta. The placenta was said to be offered to *Mami Wata*, the goddess of the sea, for personal riches. Due to this offering, people feared that they would lose their new-born baby before the age of 5 years (Table 3). These rumours substantially affected the placenta biopsy collection of the trial. The intensity of these concerns and rumours gradually grew and intensified with the progression of the trial.

#### Occurrence of an adverse event

The impact of these rumours on the trial grew after the death of a baby whose mother was enrolled in the trial. Before delivery the pregnant woman was referred to a higher level of health care (the district health centre located in Abomey-Calavi) since complications were expected. However, the pregnant woman and her relatives choose not to follow the referral advice and decided to attend a different peripheral health centre where the delivery was assisted by a less qualified auxiliary nurse in absence of the midwife. When the research team arrived at the health facility to collect the placenta biopsy, health staff had already given the placenta to the family. The trial team requested the placenta for a biopsy and performed the biopsy without the parents observing the procedure. However, when family members saw the placenta in the hands of the trial team this caused anger and a threatening crowd came running to the facility.

#### Ending of placental biopsies

Due to this unrest, the trial investigators decided to stop collecting placenta biopsies in Benin and continue to with the collection of data on secondary outcomes. Several meetings were held with health authorities (The National Malaria Control Disease (PNLP) and the Minister of Public Health) as well as with representatives of the 18 trial communities. In order to increase participation levels, door-to-door sensitizations were done by the CHWs to inform community members that placenta biopsy was not required anymore. Door-to-door sensitizations proved successful in certain villages but in others people remained sceptical, questioning the sudden change in protocol. The change in protocol confirmed people’s fear: why this sudden change when the biopsy was so important at the start of the trial? Consequently, recruitment remained low, and the trial ended prematurely in Benin on the 30th of September 2015.

### Underlying contributing factors of the rumours in Benin

#### Placenta beliefs and rituals

Among different ethnic groups in all three countries, the placenta is sacred and symbolically represents as well as determines the future of the new-born (Table 3). It is seen as part of the infant and rituals are carried out to prevent this sacred part to be used to harm the child.

In Benin, there are differences in the rituals between families but a common feature is the requirement of storing the complete placenta in a humid place to stay ‘fresh’. When placed in a ‘hot place’, the child is expected to become a ‘hot’ choleric person inclined to delinquency, further impacting the well-being of the entire family. Interestingly, similar beliefs and rituals about the link between placenta and the child’s well-being are also common among the ethnic groups participating in the trial in Burkina Faso where the placenta is called *sagado* or *regedo* (a piece of dirt) and buried in a pot in places considered to be ‘dirty’ (e.g. bathrooms, garbage dump) as contamination by water or ants would give the child ear pain. In The Gambia, some ethnic groups burry the placenta inside the compound in order to prevent the use by evil spirits. Rituals regarding placenta in The Gambia are carried out by women.

#### Uncertainty and the implementation of informed consent process

Although the research team invested substantially in the informed consent process, several problems were related to miscommunication. First, during the initial community sensitization meetings the study team translated ‘the analysis of the placenta’ in the local language as ‘preparing’ the placenta (Table 3). In the local language, the term ‘cook’ refers to medical ‘analysis’ and it is used by medical staff for the analysis of blood, urine or stool. Community members frequently interpreted, ‘analysing’ the placenta as ‘cooking’ the placenta. This translation gave the impression that the placenta would be placed in a ‘hot place’ thereby violating community member’s belief that the placenta should only be stored in cold places. Second, despite multiple community sensitization meetings and the informed consent procedures, the relevance of placenta biopsy still remained unclear to potential study participants, further leading to doubts regarding the intention of the researchers. Third, the individual informed consent procedure was carried out with the pregnant women at the health centre (Table 3). Despite the fact that men are in charge of the placenta and the rituals related to it, they were not involved during the consent procedure leading to further uncertainty. Last, there was a misunderstanding about the feedback of the study results. The research team was criticized by the community for not being able to provide immediate results of the placenta biopsies: according to them it was said that the research would allow them to know whether or not the baby was sick.

#### Community Health Workers’ role and relationship with community members

For the CHWs, trial activities represented an additional workload which prevented them from carrying out their daily activities and employment. Their involvement in the trial constrained their previous activity (i.e. home visits for sick children), whilst the compensation provided for this additional work load was seen as insufficient.

CHWs had an ambiguous position in relation to the trial and communities. Many CHWs were actively involved in political activities, which prevented the sensitization and engagement of community members supporting a different political party (Table 3). In general, politicians in the area were distrusted as they were accused to use all possible means (including occult practices) to achieve their objectives. These tensions were more outspoken during the implementation of the trial due to upcoming political elections: some communities believed that placenta biopsies would be used by the president to renew his mandate in office. The fact that CHWs often had a higher socio-economic status than most community members increased the distrust.

#### Health system factors

The governmental health care, which at community level provided e.g. vaccination or medicines’ distribution for free, was generally distrusted by the communities (Table 3). A common expression in the study area was that ‘*nothing is for free, something is always hidden behind it’*. There were previous negative experiences (including the occurrence of an adverse events after a mass drug administration) with governmental health interventions in the study area. Consequently, the free medication and health services offered as part of the COSMIC trial caused concerns among local communities.

#### Actors in generating and spreading the rumours

At the beginning of the study, the public maternity services were asked to contribute to the study by helping with participant’s recruitment and providing ANC services. For this purpose, an agreement between the local research institution and the public health services was signed. However, during the implementation of the trial, the relationships between some local partners deteriorated. Pregnant women enrolled in the trial received services and medication free-of-charge, while several midwives normally earned some pocket money by selling drugs in case of stock outs. These difficulties led to a general dissatisfaction, contributing to the generation and spread of rumours by the government health staff (Table 3). Moreover, also private health practitioners were involved in the spread of rumours in order to discourage community members to attend government health services and instead visit their private health facilities.

## Discussion

This comparative anthropological study on the implementation of a malaria in pregnancy trial in The Gambia, Benin and Burkina Faso focused on factors contributing to the premature ending of CSST trial in Benin due to rumours of placenta-selling. Considering the fact that the placenta occupies a key position in giving meaning surrounding birth in all three countries, these ‘beliefs’ did not provide an adequate explanation for the emergence and spread of the concrete rumours that affected the trail in Benin. Instead, the confluence of socio-political power relations, economic inequality, socio-cultural dynamics, informed consent procedures, and a catalyzing adverse event led to the decline of the trial in Benin.

Fears related to the collection of bodily substances and/or medication received in clinical trials are common [2, 5], and mostly reflected in the fear of blood selling, trade of body parts, sterilization campaigns and the deliberate spreading of diseases. Sensitivity around the placenta was, therefore, to be expected in the three trial sites.

Determining underlying additional factors, however, were not accounted for or foreseen in the trail set up. The already existing distrust towards the current government, contributed to the generation of rumours due to the close collaboration of the trial with the ministry of health and the political nature of some of the—party affiliated—CHWs. Existing socio-economic inequality apparent, for example, in the relatively better living conditions of some CHWs, was an additional key element acted out through the placenta-selling accusation, as has been shown to be the case for sorcery accusations across sub-Saharan African countries [24–26]. As such, the incentives the CHWs received as remuneration for their work in the trial, were interpreted as *‘*the price of the placenta’ by other community members. Furthermore, the ambiguity created during the informed consent process, related to the challenging translation of technical terms allowed for misinterpretation. This raises the importance of translation in medical research, especially when they are carried out among population with low literacy rate and with various dialects [27]. Correct translations might be costly in terms of time and money, but should be considered while developing the research proposal and intervention. Less obvious, however, was the fact that the informed consent process did not target men in the community who, nevertheless, were traditionally key in handling the placenta after birth in Benin unlike the defined female responsibilities in the research settings in The Gambia and Burkina Faso. As could be expected women were targeted in a pregnancy trial and not their husbands.

The added value of community engagement, for example in the form of community sensitizations and Community Advisory Boards (CAB), has been suggested in order to allow (i) appropriate translation and implementation of informed consent procedures and (ii) improve mutual understanding of research purposes between research teams and local communities [3, 27]. ﻿It should be taken into account that engagement with community members always implies engaging with existing social relations and hierarchies and careful considerations should be made regarding the representativeness of communities [3, 28–32].

This analysis highlights that rumours are not the sole reflection of ignorance, to be solved through ‘community awareness’ campaigns [4]. In this context, the community awareness campaign, designed to tackle the rumours, actually achieved the opposite effect in several communities, i.e. adding further suspicions to the original intent of placenta sampling. When looking at the main actors in the spread of the rumours, the critical importance of health staff became apparent: CHWs demanding higher incentives and midwives’ lack of income from privately sold drugs during ANC. These actors and related social dynamics highlight that more directed strategies are required beyond targeting ‘the community’ with additional ‘knowledge’.

## Conclusion

Specific transdisciplinary social science research designs, accompanying the implementation of the trial, and integrating multiple stakeholders’ knowledge and involvement are required to define and solve upcoming barriers. In addition to community-based ethnographic research and emergent theory approaches, communities should be involved as co-creators of the intervention and, as far as possible, as co-implementers involved. Through dialogical engagement, communities can become creative counterparts in the definition of explanations and the search of solutions towards particular goals: they can share ideas to co-develop the project strategy. Even though these discussions are highly focused on potential and emerging barriers, dialogic engagement provides tools to contextualize programmatic expectations based on the reality of involved populations.
